# Supplementary material for: MR. Estimator, a toolbox to determine intrinsic timescales from subsampled spiking activity
Source: PLoS One. 2021 Apr 29;16(4):e0249447. doi: 10.1371/journal.pone.0249447 (PMC8084202; doi:10.1371/journal.pone.0249447)
Supplement: Attachment — Submitted filename: Response to Reviewers.pdf [file pone.0249447.s001.pdf]

“MR. Estimator, a toolbox to determine intrinsic timescales from subsampled spiking activity”

Dear Editors,
Dear Reviewer,

We thank the reviewer for the thoughtful comments and positive feedback. We made changes and additions according to the suggestions, and now include a real-world example in the appendix.

We want to address the suggestions point by point, below.

> Reviewer #1: In this manuscript, Spitzner et al. introduce a Python package to extract intrinsic timescales from data that is well described by a subsampled AR process of first order. To this end, the package relies on the multiple-step regression method developed in (Wilting & Priesemann 2018). Beyond introducing the package and recapitulating the method, the authors investigate the bias due to short trials and derive an analytical approximation thereof. Given the broad applicability of AR processes and the ubiquity of the subsampling problem, it is certainly a valuable contribution - with potential impact across multiple disciplines - to provide an easily usable, well tested, and open source reference implementation.
>
> The manuscript is well written and on point. Maybe, more importantly, the user-level documentation of the package itself is excellent. After reading the paper and small parts of the documentation, it was straightforward to install the package and to use it on custom problems. On these problems, the package yielded the correct results and it seemed efficient although I did not perform any rigorous testing / profiling. Lastly, the code is well structured and documented.

Thank you very much for the kind words.

> I have only a few minor comments / remarks:
>
> 1. I think it would be good to include a ‘real-world’ use case to demonstrate the utility of the package.

Thank you for this suggestion.
We now analyze a real-world example in Sec. 3 and provide the code in the appendix.
The example uses openly available spiking data from rat hippocampus during an open field task, where, in addition to the intrinsic timescale, the toolbox correctly infers the frequency of theta oscillations that are characteristic for the recording.

> 1. What is the difference between the multi-step regression and an estimate of the normalized connected correlation function? Below Eq. (3) both approaches are called similar, below Eq. (5) it is stated that they are equivalent in the stationary case, but their difference is not discussed as far as I can see.

We revised the entire section to make it clearer.
The two approaches are almost identical in the sense that both fit an estimate of the correlation function.
For the multi-step regression estimate, the correlation coefficients $r_k = \mathrm{Cov}[A_t, A_{t+k}]/\mathrm{Var}[A_t]$ are fitted.
They directly relate to the branching parameter (and for stationary activity to the autocorrelation time) as

we now derive more clearly in the text. This is different from the time-dependent Pearson correlation coefficient defined as $C(A_t, A_{t+k}) = \mathrm{Cov}[\mathrm{A_t}, \mathrm{A_{t+k}}]/\mathrm{Std}[\mathrm{A_t}]\mathrm{Std}[\mathrm{A_{t+k}}]$ — which is often done, but requires time-translational invariance to infer the branching parameter.

> 1. The role of stationarity in section 4.1 is not entirely clear to me. I think Eqs. (5–12) all assume stationarity. If this is correct I suggest to state it explicitly. On first reading, the “special case” seemed to apply only to Eq. (5) but not to the remainder of the section.

Indeed, we did not state clearly when the stationarity assumption was made. We now explicitly say at which point we assume stationary activity and restructured the section so that it should be clear that everything that follows keeps the assumption.

> 1. Please define both “intrinsic timescale” and “autocorrelation time” in the introduction to clarify in which context they are not synonymous.

We now mention in the introduction that both are synonymous in this work.
Having said so, we do not know of an example where they are *not synonymous*. Rather, the “intrinsic timescale” is a term that represents a general concept and is often quoted even when a precise definition is not given. However, to our knowledge, whenever a definition is given, it matches that of the autocorrelation time.

> 1. Would it be sensible to use a logarithmic y-axis in Fig. 2 to visualize the invariance of the timescale?

We added this as a second panel and annotated the slope.

> 1. The title of section 4.1 seems at mismatch with the content. Furthermore, this section is very dense and it might be worthwhile to expand it slightly and motivate the various manipulations a bit more.

We renamed the section to (hopefully) better match the content and added more motivation of subsequent steps. The new title is *“Derivation of the multi-step regression estimator for autoregressive processes”*

> 1. Section 3 is not included in the overview at the end of the introduction.

We added it to the list, and also mention that it includes the real-world example.

> 1. Is there a particular reason why \sum_i^N is used instead of \sum_{i=1}^{N}, e.g. in Eq. (16)?

We corrected it to \sum_{i=1}^{N}

> 1. Figure 1 looks corrupted (black background, labels almost invisible) in the PDF but not when downloaded as a tiff file.

Thank you for reporting this issue.
This seemed to arise from file conversion (we were not allowed to provide the compiled pdf). We will discuss it with the Editor.
